# Research Priorities on the Relationship between Wasting and Stunting

**DOI:** 10.1371/journal.pone.0153221

**Published:** 2016-05-09

**Authors:** Chloe Angood, Tanya Khara, Carmel Dolan, James A. Berkley, WaSt Technical Interest Group

**Affiliations:** 1 ENN, Oxford, Oxfordshire, United Kingdom; 2 KEMRI-Wellcome Trust Research Programme, Kilifi, Kenya; 3 Centre for Tropical Medicine & Global Health, Nuffield Department of Medicine, University of Oxford, Oxford, Oxfordshire, United Kingdom; The Hospital for Sick Children, CANADA

## Abstract

**Background:**

Wasting and stunting are global public health problems that frequently co-exist. However, they are usually separated in terms of policy, guidance, programming and financing. Though both wasting and stunting are manifestations of undernutrition caused by disease and poor diet, there are critical gaps in our understanding of the physiological relationship between them, and how interventions for one may affect the other. The aim of this exercise was to establish research priorities in the relationships between wasting and stunting to guide future research investments.

**Methods and Findings:**

We used the CHNRI (Child Health and Nutrition Research Initiative) methodology for setting research priorities in health. We utilised a group of experts in nutrition, growth and child health to prioritise 30 research questions against three criteria (answerability, usefulness and impact) using an online survey. Eighteen of 25 (72%) experts took part and prioritised research directly related to programming, particularly at the public health level. The highest-rated questions were: “Can interventions outside of the 1000 days, e.g. pre-school, school age and adolescence, lead to catch-up in height and in other developmental markers?”; “What timely interventions work to mitigate seasonal peaks in both wasting and stunting?”; and “What is the optimal formulation of ready-to-use foods to promote optimal ponderal growth and also support linear growth during and after recovery from severe acute malnutrition?” There was a high level of agreement between experts, particularly for the highest ranking questions.

**Conclusions:**

Increased commitment to rigorous evaluations of treatment and prevention interventions at the public health level, addressing questions of the timing of intervention, and the extent to which impacts for both wasting and stunting can be achieved, is needed to inform global efforts to tackle undernutrition and its consequences.

## Introduction

Wasting and stunting are priority global public health problems. It is estimated that wasting affects 52 million children (19 million with severe wasting) and stunting affects 165 million children under five.[1] Each year, approximately 800,000 deaths are attributed to wasting (60% of which are attributable to severe wasting) and over one million to stunting.[2] Wasting and stunting are also associated with the loss of 64.6 and 54.9 million Disability Adjusted Life Years (DALYs) respectively, accounting for 14.8% and 12.6% of the total global DALYs for children under five.[3]

Globally, improvements in levels of stunting and wasting have been achieved. The prevalence of global under-five stunting has decreased from 40% in 1990 to 32% in 2005 to 25% in 2011. The greatest gains have been made in Asia, where stunting prevalence is highest (from 188.7 million in 1990 to 98.4 million in 2010), and in Latin America and the Caribbean (13.7 million in 1990 to 7.4 million in 2010).[2] The global prevalence of wasting in under-fives has also decreased over the last twenty years, by 11% since 1990. Again, the highest prevalence of wasting is in Asia, where 70% of the world’s wasted children live. However, in spite of these recent gains, projections show that the current World Health Assembly (WHA) wasting and stunting targets will not be met at the current rate of improvement.[4, 5]

The Lancet’s Maternal and Child Nutrition series in 2008[3] and 2013[2] identified a number of evidence-based specific nutrition interventions focusing both on treatment and prevention of some of the immediate and underlying causes of undernutrition, which if scaled up could reduce undernutrition-related mortality and disease burden by an estimated 25% in the short term. Increased attention to nutrition sensitive interventions that aim to prevent undernutrition by targeting basic and underlying causes was highlighted as a priority to meet global nutrition targets. A recent paper on the WHO 2025 stunting targets also highlights the need for nutrition-sensitive development, and the development of multi-sectoral plans to address stunting at national scale, as well as an accountability framework and surveillance systems to monitor progress.[5] This policy direction has been taken on by the Scaling Up Nutrition (SUN) movement as well as other global programmes and research and financing initiatives.

In parallel to these developments, a number of reviews[6–10] have highlighted the separation between wasting and stunting in the architecture of nutrition at policy, financing, programme and research levels and suggested that addressing this separation may also be important to achieving global nutrition targets.

“The fragmentation of interests and perspectives on childhood undernutrition has negative consequences for advocacy efforts that aim to bring attention and resources to child nutrition across the globe. It also has serious implications for how children worldwide receive nutrition interventions and services.” (Menon & Stoltzfus, 2012)^7^

Both stunting and wasting are present in the majority of developing countries and contexts (something also highlighted in the 2014 Global Nutrition Report);[5] can occur concurrently in the same child (highlighted in the 2015 Global Nutrition Report);[11] and share underlying and basic causal factors such as infectious diseases, environmental enteric dysfunction, a diet with inadequate nutrients, and suboptimal infant feeding and caring practices. As a result, there is a need for the international community to look more closely at the way children experience these two manifestations of undernutrition over time, and focus programming (particularly prevention approaches) and research more coherently towards achieving impacts for both.

This exercise aims to inform the research agenda of governments, researchers, donors, international agencies and national agencies about questions that are most likely to result in high impact policies and practices that address the links between wasting and stunting.

### Ethics statement

As is standard for CHNRI projects, formal ethics review was not needed since the work does not involve any personal or otherwise sensitive data and works with professional participants rather than patients. Participants were solicited via established professional networks, whose key purpose is to facilitate and enable information-sharing and ‘group activities—such as CHRNI. Participants were aware that their responses would be used for research. Those who completed the questionnaire were asked whether they were happy to be named as part of the ‘MAMI group author’ list and only those answering “yes” are listed. Individual answers to the questions are not presented and are anonymous.

## Methods

We used the Child Health and Nutrition Research Initiative (CHNRI) methodology for setting research priorities in health, described in detail elsewhere.[12–15] This is a well-recognised and tested methodology for setting research priorities that enables the systematic and transparent development, listing and scoring of possible research questions against pre-defined criteria.[16] It enables individual experts to weigh up the strengths and weaknesses of research options and, as a group, to produce a list of research questions in which investments should be prioritised. The results have the potential to provide a powerful advocacy tool for investment in research from international agencies, research funding bodies, donors, governments and policy-makers.[15]

We first defined the context and criteria for priority setting, displayed in Box 1. We agreed to use an existing Technical Interest Group (TIG) facilitated by ENN as the expert group. The TIG had been set up at the beginning of 2014 to guide and contribute to a review of the relationship between wasting and stunting. It is made up of 25 individuals with extensive experience in research and programming for wasting and stunting and represents expertise with different perspectives from a range of academic institutions, UN bodies, donors and NGOs. The TIG identified possible research questions during the process of reviewing and discussing the relationship between wasting and stunting in 2014 through email and face to face exchanges.[10]

**Box 1. Context and criteria of the CHNRI exercise on wasting and stunting.**

**Burden of disease of interest:** Wasting affects 52 million (19 million severe wasting) and stunting 165 million under 5’s each year. Estimates for the deaths attributable to wasting based on these prevalence figures is 875,000/yr (516,000 of these are attributable to severe wasting) and for stunting 1 million (15% of overall chid deaths). Stunting is associated with 54.9 million DALYs (12.6% of the total global DALYs for children under 5) and Wasting with 64.6 million DALYs (14.8% of the total global DALYs for children under 5).

**Population of interest:** All developing countries with a burden of stunting and wasting, individuals within the 1000 days and beyond.

**Existing policy/target:** WHA targets: Wasting—reduce and maintain prevalence of childhood wasting to <5%, Stunting—Reduce by 40% the number of under-five children who are stunted. **Level of urgency:** The level of urgency is always high where there is an elevated risk of excess infant and child mortality associated with wasting and stunting.

**Time frame:** To achieve measurable improvements in the burden of wasting and stunting within 5 years.

CHNRI methodology recognises that the number of possible health research options is “endless and limited only by imagination of all living researchers”. It is not therefore possible to achieve a list of all possible options, however, to achieve the widest possible list of options, CHNRI methodology recommends the use of a theoretical framework to consider possible options against. A smaller group of TIG members, including the authors of this paper, took the list of possible research questions proposed by the wider group and placed them within the recommended theoretical framework. Questions were refined according to this framework and resulted in a list of 30 possible options. The list of 30 options located within the CHNRI theoretical framework is described in Table 1.

**Table 1:** List of research questions located in the research framework.

**Measuring the burden of wasting & stunting**	1.	How to estimate incidence of wasting over time in different contexts?
		What is the global burden of children experiencing wasting and stunting concurrently?
		What are the implications of the global burden of concurrent wasting and stunting on the global burden of mortality?
		What is the global burden of severe stunting?
		What are the implications of the global burden of severe stunting on mortality burden?
		How do wasting and stunting develop and interplay in individuals over time?
**Understanding risk factors for wasting & stunting**	2.	What is the role of pre-pregnancy nutritional status in determining risk of being born stunted and/or wasted?
		Does foetal growth (in terms of timing of deficits in ponderal and/or linear growth) predict wasting and stunting?
		Does anthropometric status at birth (in terms of ponderal and/or linear growth) predict wasting and stunting in childhood?
		Does the process of stunting (slowing of linear growth) or wasting (loss of weight) carry greater risks for a child compared to the end point of being stunted or wasted in relation to the growth reference?
		What role does gut health/inflammation play in wasting?
		What are the long-term implications of wasting and stunting (separately and combined) in early life on adult health.
**Evaluating existing interventions**	3.	What are the implications of rapid weight gain (as during wasting treatment) on body composition and function in childhood?
		What are the implications of rapid weight gain (as during wasting treatment) on body composition and function in adulthood?
		Does treatment of wasting support catch-up in linear growth?
**Basic research to develop or improve interventions**	4.	What are the physiological/functional changes which occur during wasting and stunting and when both are underway concurrently?
		How does body proportion and composition change during wasting and stunting, in particular muscle and fat mass?
		At what level of wasting does linear growth slow down or speed up?
		Does mid-upper arm circumference (MUAC) preferentially identify children for treatment who have lower weight-for-height and height-for-age in different contexts?
**Clinical research to develop or improve interventions**	5.	What is the optimal formulation of RUTF to promote optimal ponderal growth and also support linear growth during and after SAM recovery?
		Can nutrition convalescent support (e.g. provision of nutritional supplements & support triggered by a drop in weight-for-height or weight-for-age after acute illness), prevent both wasting and stunting?
		What existing interventions work for treating severe stunting in order to prevent associated mortality?
		What new interventions work in trial conditions for treating severe stunting in order to prevent associated mortality?
**Public Health research to develop or improve interventions**	6.	Can interventions outside of the 1000 days, e.g. pre-school, school age and adolescence, lead to catch-up in height and in other developmental markers?
		What existing interventions work for stunting reduction?
		What new interventions work in trial conditions for stunting reduction?
		What timely interventions work to mitigate seasonal peaks in undernutrition (both wasting and stunting)?
		What are effective packages of interventions for both maternal nutrition and new-born outcomes?
		How can pre-pregnancy nutrition support for adolescent girls be effectively and appropriately delivered?
		What practical linkages between interventions to treat and prevent wasting and stunting will have the highest impact? (e.g. referral systems between programmes, carrying out mid-upper arm circumference (MUAC) checks at routine points of contact, etc.)

CHNRI recommends a set of defined criteria with which to judge each of the research questions. We took the five basic criteria recommended in CHNRI methodology and, as recommended in the CHNRI guidelines, for the sake of simplicity, merged “effectiveness” and “deliverability” into one criterion named “usefulness”, and the criteria “maximum potential for disease burden reduction” and “effect on equity” into one criterion named “impact”.[15] We felt that it was important that the exercise was as straight forward and simple as possible, without compromising on its usefulness, in order to encourage as many experts as possible to complete the survey. Reducing the criteria to three allowed us to do this, and was explained to the expert participants to aid common interpretation of the criteria. We used the following definitions for each of the criteria:

1. *Answerability*: answerability of the research question (i.e. is it well framed, with well-defined end points and is it likely to gain ethical approval?)
2. *Usefulness*: usefulness of answering the research question (i.e. would the intervention that would be developed/ improved as a result of the research be deliverable, effective and efficacious?)
3. *Impact*: Would the successful reaching of research endpoints for this question have high impact (i.e. have the capacity to remove 5% or more of the disease burden, and be likely to most benefit the most underprivileged?)

Experts were asked to envision what future interventions arising from each research question might be in order to assess both the usefulness and impact of the specific research question. The team did not feel that further guidance for each of the criteria was required, being relatively confident that experts were familiar with the CHNRI methodology and that, as a result, there would not be too much heterogeneity in the way that criteria were applied to the research questions.

The 30 research questions were tabled against each of the three criteria and thus a total of 90 queries formed the basis of an online survey (www.surveymonkey.com). The survey was ordered so that the scorers judged all of the questions against each of the judging criteria in turn. All members of the TIG were sent emails with a link to the online survey to invite them to take part. These are included as ‘group authors’ at the end of the paper.

Experts answered the questions listed in Table 1 by selecting either ‘Yes’ (valued as 1 point), ‘No’ (0 points), ‘Undecided’ (0.5 points) or ‘Insufficiently informed’ (defined as ‘missing input’). Each of the 30 research options received three scores, one for each criterion, each ranging between 0% and 100%. The overall research priority score (RPS) was calculated as the mean of all three priority scores and the priority of research questions were ranked accordingly.

The ‘Average Expert Agreement’ score (AEA) was also computed, as recommended by CHNRI.[15] The AEA provides a measure of the level of agreement or controversy between expert scores in the answer given. This was calculated for each research option in our survey as follows [14]:

$$AEA=\frac{1}{9}\sum_{q=1}^{9}\frac{N(scores\;providing\;the\;most\;frequent\;response)}{N(scores\;providing\;any\;response)}$$ (1)

(where q is a question that experts are being asked to evaluate competing research investment options, ranging from 1 to 3).

Some CHNRI surveys have applied weightings to the different judging criteria used, according to the values of a wider group of stakeholders, however, the majority of CHNRI exercises have omitted this step. We decided to also omit this stage as we felt that the three criteria were equally important.

## Findings

Eighteen (72%) of the 25 TIG members took part in the survey. Sixteen completed the survey in full. Respondents were from a mix of academic institutions, NGOs and UN agencies. Most were academics (N = 10), some were engaged in operations/programming (N = 3), one was engaged primarily in policy and others declared their involvement in a mix of these activities (N = 4).

The range of overall research priority score (RPS) was 46.7 to 91.1, and for the top 25 questions, RPS ranged from 69.8 to 91.1. Table 2 shows the top ten ranked research questions. The highest ranking research question was “Can interventions outside of the 1000 days, e.g. pre-school, school age and adolescence, lead to catch-up in height and in other developmental markers?” This question scored very highly against all three judging criteria. The second highest scoring question was, “What timely interventions work to mitigate seasonal peaks in undernutrition (both wasting and stunting)?” which scored particularly highly against ‘answerability’. The third highest ranking question was, “What is the optimal formulation of RUTF to promote optimal ponderal growth and also support linear growth during and after SAM recovery?” which scored particularly highly against the criteria ‘usefulness’. The fourth highest ranked question was “What is the role of pre-pregnancy nutritional status in determining risk of being born stunted and/or wasted?” The fifth highest was, “What are effective packages of interventions for both maternal nutrition and new-born outcomes?”

**Table 2:** Top ten questions ranked by RPS.

Question	Rank	Question no.	Answerability	Usefulness	Impact	RPS	AEA
**Can interventions outside of the 1000 days lead to catch-up in height and in other developmental markers?**	1	24	93.8	93.8	85.7	**91.1**	0.84
**What timely interventions work to mitigate seasonal peaks in undernutrition (both wasting and stunting)?**	2	27	93.3	86.7	86.7	**88.9**	0.80
**What is the optimal formulation of RUTF to promote optimal ponderal growth and also support linear growth during and after SAM recovery?**	3	20	84.4	93.8	83.3	**87.2**	0.85
**What is the role of pre-pregnancy nutritional status in determining risk of being born stunted and/or wasted?**	4	7	88.2	86.7	85.7	**86.9**	0.82
**What are effective packages of interventions for both maternal nutrition and new-born outcomes?**	5	28	93.3	90.6	75.0	**86.3**	0.79
**Can nutrition convalescent support prevent both wasting and stunting?**	6	21	89.3	90.6	73.3	**84.4**	0.79
**What practical linkages between interventions to treat and prevent wasting and stunting will have the highest impact?**	7	30	78.1	90.6	83.3	**84.0**	0.74
**What new interventions work in trial conditions for treating severe stunting in order to prevent associated mortality?**	8	23	71.9	87.5	89.3	**82.9**	0.74
**What role does gut health/inflammation play in wasting?**	9	11	81.3	90.6	76.7	**82.8**	0.72
**Does treatment of wasting support catch-up in linear growth?**	10	5	86.1	78.1	80.0	**81.4**	0.77

All of the top three questions were categorised under the research instrument ‘research for the development of new interventions/to improve existing interventions’, as indeed were eight out of the top ten research questions. This demonstrated the group prioritised research that directly relates to programming and public health, rather than epidemiological research. Most would involve testing interventions at the public health level, such as interventions outside of the first 1000 days (Q24), interventions to mitigate seasonal impacts (Q27), interventions that use Ready-to-Use Foods (Q20), improved nutritional convalescent support (Q21) and identifying synergies between interventions (Q28 and Q30).

**Fig 1:**
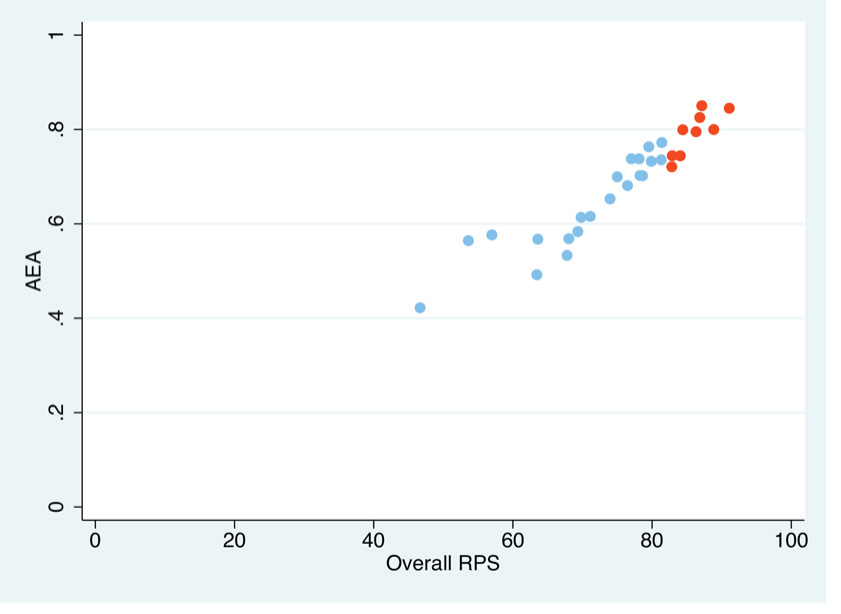
Scatterplot of AEA vs. RPS for all questions, with the top 10 by RPS shown in red.

The average expert agreement score (AEA) ranged from 0.42 to 0.85 overall, with the top ten scoring questions having a high AEA (>0.7), and progressively less agreement amongst lower scoring questions (Fig 1, linear regression of AEA against rps, P<0.001). Lower AEA was observed for questions with a modal response of 0, indicating that no questions were universally rejected by the expert panel, perhaps reflecting the pre-screening of questions. However, this also reflects less agreement about what not to study from the 30 selected questions, which is a typical pattern seen with CHNRI studies, reflecting the different interests of the expert participants.

Importantly, this group of experts agree that the top six research questions should be given the greatest priority (with the highest level of agreement being for question 20, “What is the optimal formulation of RUTF to promote optimal ponderal growth and also support linear growth during and after SAM recovery?”, ranked third by RPS).

## Discussion

This CHNRI research prioritisation exercise shows that research that directly relates to interventions and programming, rather than purely observational research, should be prioritised. This prioritisation reflects the complexity of underlying and proximate causes of wasting and stunting, meaning that outcomes cannot necessarily be predicted from simply observational research. Taking this forward will require increased commitment from funders, academics and implementing agencies to undertake clinical trials or large-scale programmatic evaluations that are rigorous in design, with appropriate sample size, choice of comparator groups and follow up for meaningful health outcomes.

The highest-rated question, “Can interventions outside of the 1000 days, e.g. pre-school, school age and adolescence, lead to catch-up in height and in other developmental markers?” arises from evidence of opportunities for catch-up growth in older age groups (i.e. outside the first 1000 days) particularly during adolescence.[17] It is an area that has had little focus but could have important implications, particularly in the case of adolescent girls, as evidence shows that maternal stature may be a predictor of her child’s size at birth.[2] The timing of interventions to promote catch up growth in mid-childhood and adolescence is not well understood but may be important; studies of immigrant populations have shown that childhood catch up growth can increase risk of early pubertal development, which can abbreviate the childhood growth period and limit final height.[18] Though the 1000 days message has been an important one to galvanise action, the group identified further investigation of these other lifecycle opportunities, particularly supporting adolescent growth, as potentially key for meeting undernutrition targets. Though the question of the timing of catch-up from stunting refers specifically to stunting, rather than to the relationship between stunting and wasting, it was not surprising to the authors that it received top ranking. The question of different age target groups for wasting (<5’s) and stunting (<2’s) programming was at the time the subject of a number of high profile articles and debates at conferences as well as within the TIG, and is critical to programming.

The second question, “what timely interventions work to mitigate seasonal peaks in undernutrition (both wasting and stunting)?” relates to strong seasonal patterns of stunting and wasting, illustrated in a number of countries.[19, 20] These patterns may be linked to seasonality in the causal factors and/or may even illustrate a degree of direct correlation between wasting and stunting–this is an area where many questions still remain unanswered. However, recent trials, such as the provision of seasonal nutritional supplementation in Niger,[21] have shown effects on both wasting and stunting. Further studies are underway in this area, including into the role of cash and/or voucher transfers in preventing seasonal increases in wasting.[22] The prioritisation of this question is a call for future studies to measure effects on both stunting and wasting.

The third question, “what is the optimal formulation of RUTF to promote optimal ponderal growth and also support linear growth during and after SAM recovery?” is underpinned by the fact that only a few studies of SAM or MAM treatment have looked at linear growth during or after treatment, or compared different formulations of RUTF in trials of adequate size. Those studies that have suggest that there is no positive effect of RUTF based treatments on linear growth outcomes. [10, 23] However, there is some evidence that linear growth ceases or slows during periods of wasting[24–27] and therefore, the timing of restarting of linear growth and how this might be supported during wasting treatment is an area deemed important for more focussed research.

The fourth and fifth questions, “what is the role of pre-pregnancy nutritional status in determining risk of being born stunted and/or wasted?” and “what are effective packages of interventions for both maternal nutrition and new-born outcomes?” relate to the highest-rated question. There is some evidence that suggests that pre-pregnancy maternal nutritional status in terms of stature, BMI, and micronutrient status, may play a role in determining length and weight at birth.[2] This does not negate the need to support maternal nutrition during pregnancy but suggests that they may be important opportunities to support nutrition pre-pregnancy that are not currently being capitalised on. The intergenerational cycle of growth failure links small maternal size back to mothers’ growth in childhood and adolescence and even back to her own size at birth. This suggests that pre-pregnancy nutritional status may be extremely important for determining the risk of a child being born with linear and/or ponderal growth deficits. Therefore, there is increasingly a call for focus on pre-conceptional interventions for improving maternal pre-pregnancy BMI, and interventions that can influence linear growth and attained adult height in order to benefit foetal growth.[28] Further investigating the origins of wasting and stunting could help to strengthen support for maternal nutrition, and help programmers to expand their windows of opportunity to break the intergenerational cycle of undernutrition.

In general, there remains a critical need for clinical trials and evaluation to be conducted rigorously in programmatic contexts, with adequate sample sizes and standardised designs that assess outcomes both for wasting and stunting in order to identify the potential for approaches to influence both public health problems. In a recent review of trials of supplementary feeding interventions, the considerable heterogeneity between studies made it difficult to draw firm conclusions.[29] These considerations also apply to more development-oriented approaches, such as positive deviance/hearth or water and sanitation interventions, where designs, methodological quality and results have been variable.[30, 31]

Increasingly, the focus of international nutrition is on multi-sectoral interventions (involving health education, agriculture, social, political and environmental actors in both the humanitarian and development domains), particularly for the prevention of undernutrition. The more such multi-sectoral approaches can be assessed in terms of impact on both stunting and wasting, the better their contribution to achieving country and global nutrition targets can be evaluated.

### Strengths and limitations

The CHNRI methodology proved to be a helpful tool for systematically listing and scoring specific research questions in a transparent way against specific criteria. It allowed experts to score questions independently of each other and therefore limited the influence of individuals that might express strong opinions in a group setting. The CHNRI methodology also generated quantitative outputs that were relatively easy for the group to interpret, including clearly ranked research priorities, and a measure of the questions that cause the greatest agreement and controversy.

Our study has some weaknesses. A relatively small number of experts were engaged in the process of setting research questions (N = 25) and an even smaller number in the priority setting exercise (N = 18). This may expose the results to the biases of this particular group (e.g. most respondents were academics from Western institutions) and may mean that scores are not stable. However, the pool of experts in this area globally is relatively small, and a high level of engagement was required for both the review and research prioritisation, which made it difficult to recruit a larger number of individuals into the TIG. Results can be seen as a useful guide for research investments in this important area nonetheless. Furthermore, not all possible research options were included in the study, nor could they be whilst maintaining a feasible methodology. In future, other research options, not included here, are likely to arise given the interest in this area and rapidly emerging evidence on stunting in particular. This limits the applicability of these results as time passes. In this case, a repetition of the exercise would be required. In addition, the research options considered were those that would be likely to achieve impact on the burden of wasting and stunting within five years (Box 1). This may have influenced the type of research options chosen by limiting research options that would take a longer time to deliver results. We chose to limit the potential scope of studies in this way in order to maintain focus to the exercise, but also given that this is a realistic time frame to expect research donors to fund. Finally, as we decided to omit the stage of the CHNRI process that applies weightings to each of the judging criteria based on a wider set of societal values, as we felt all criteria were of equal importance. We therefore perhaps missed an opportunity to engage a wider audience and to reflect societal values in the results of the study. Nevertheless, we believe these results to be an important guide in the selection of research options for investment.

### Conclusions

The need to reduce current levels of wasting and stunting is compelling and is reflected in the WHA targets and in the emerging SDGs. There is unprecedented global momentum to drive levels of undernutrition down and many country governments are putting in place policies and programmes to achieve these reductions. However, the current degree of separation between wasting and stunting policy, guidance, programming and financing, may hinder the pace of change required and the evidence, though weak, does suggest that preventative approaches should target both forms of undernutrition simultaneously. New evidence, especially for stunting, is rapidly emerging, thus the CHNRI process is likely to need updating over time, as in other research areas. High priority research questions are currently those that trial or study interventions, mostly (though not exclusively) at the public health level that can inform the appropriate timing of treatment and prevention interventions and the extent to which impacts for both wasting and stunting can be achieved. As in other fields related to child development, health and survival, it is essential that high quality evidence from rigorous evaluations is generated in order to inform global efforts to tackle the ongoing high burdens of wasting and stunting.

## Abbreviations

AEA: Average Expert Agreement
CHNRI: Child Health and Nutrition Research Initiative
DALYs: Disability Adjusted Life Years
MDGs: Millennium Development Goals
NGO: Non-governmental organisation
RPS: Research Priority Scores
RUTF: Ready-to-use Therapeutic Food
SAM: Severe Acute Malnutrition
SDGs: Sustainable Development Goals
TIG: Technical Interest Group
UN: United Nations
WHA: World Health Assembly
